# Adenosine Receptor-Mediated Cardioprotection—Current Limitations and Future Directions

**DOI:** 10.3389/fphar.2018.00310

**Published:** 2018-04-04

**Authors:** Robert D. Lasley

**Affiliations:** Department of Physiology, Wayne State University School of Medicine, Detroit, MI, United States

**Keywords:** adenosine receptor subtypes, cardioprotection, chronic myocardial ischemia, co-morbidities, clinical trials

## Abstract

Since the seminal reports of adenosine receptor-mediated cardioprotection in the early 1990s, there have been a multitude of such reports in various species and preparations. Original observations of the beneficial effects of A_1_ receptor agonists have been followed up with numerous reports also implicating A_2A_, A_3_, and most recently A_2B_, receptor agonists as cardioprotective agents. Although adenosine has been approved for clinical use in the United States for the treatment of supraventricular tachycardia and coronary artery imaging, and the selective A_2A_ agonist, regadenoson, for the latter, clinical use of adenosine receptor agonists for protecting the ischemic heart has not advanced beyond early trials. An examination of the literature indicates that existing experimental studies have several limitations in terms of clinical relevance, as well as lacking incorporation of recent new insights into adenosine receptor signaling. Such deficiencies include the lack of experimental studies in models that most closely mimic human cardiovascular disease. In addition, there have been very few studies in chronic models of myocardial ischemia, where limiting myocardial remodeling and heart failure, not reduction of infarct size, are the primary endpoints. Despite an increasing number of reports of the beneficial effects of adenosine receptor antagonists, not agonists, in chronic diseases, this idea has not been well-studied in experimental myocardial ischemia. There have also been few studies examining adenosine receptor subtype interactions as well as receptor heterodimerization. The purpose of this Perspective article is to discuss these deficiencies to highlight future directions of research in the field of adenosine receptor-mediated protection of ischemic myocardium.

Although the hypothesis that adenosine could be cardioprotective first became recognized in the early to mid-1980s, it started inauspiciously. Based on observations that the post-ischemic heart was characterized by both decreased ATP content and reduced ventricular function, Reibel and Rovetto (1978) reported that a reperfusion infusion of adenosine (50 μM) in isolated perfused rat hearts did not improve ATP content, although ventricular function was not measured (Reibel and Rovetto, 1978). Several subsequent studies did yield beneficial effects of exogenous and endogenous adenosine on post-ischemic ATP content and ventricular function, but these results were based in large part on initiating treatment prior to the onset of ischemia (Humphrey and Seelye, 1982; Dhasmana et al., 1983; Ely et al., 1985). Initial reports that reperfusion infusions of adenosine could reduce infarct size (Olafsson et al., 1987; Pitarys et al., 1991), could not be replicated in subsequent studies (Homeister et al., 1990; Goto et al., 1991; Vander Heide and Reimer, 1996). Interest in adenosine's effects in protecting the ischemic heart did not gain widespread acceptance until early 1990 when our laboratory first reported an A_1_ receptor cardioprotective effect (Lasley et al., 1990). A year later Liu et al. (1991) reported a role for A_1_ receptors in ischemic preconditioning to reduce myocardial infarct size. Over the next 30 years there has been an explosion in the number of adenosine receptor cardioprotection studies, primarily focusing on infarct size reduction, with all four adenosine receptor subtypes (A_1_, A_2A_, A_3_, A_2B_) being implicated (Headrick and Lasley, 2009; McIntosh and Lasley, 2012).

Despite these numerous reports in experimental models, there have been few, if any, clinical trials of adenosine receptor cardioprotection in humans. Searching the database at “ClinicalTrials.gov” with the terms “ischemic heart disease” and “adenosine” yields 192 trials; changing the disease/condition to myocardial infarction (MI) yields only 40 trials. Nearly all of these trials have related to adenosine's effects on arrhythmias, coronary blood flow, and platelets. When searching this database with “adenosine receptor” 582 trials are shown, but combining with “myocardial infarction” only 9 trials are cited, and the majority of these trials used adenosine, not a receptor analog thereof. The only adenosine receptor agonists used in cardiac related clinical trials have been the A_1_ agonists Selodenoson (DTI-0009) and Capadenoson (BAY68-4986) for controlling ventricular rate in atrial fibrillation, the A_2A_ agonist regadeson and related agents for coronary imaging, and most recently the partial A_1_ agonist Neladenoson bialanate (BAY1067197) for heart failure.

This limited translation of hundreds of experimental studies, in every animal species tested to date, to the clinical arena raises the question of whether the past 30 years of adenosine receptor cardioprotection investigations have been for naught. This perspective will examine limitations of our existing knowledge of adenosine-mediated protection of the ischemic heart to help guide future studies to fully understand the cardioprotective effects of adenosine therapeutics and harness its potential in humans.

## Lack of clinically relevant animal models

Nearly all experimental studies on adenosine receptor cardioprotection have been conducted in normal, healthy adult animals. In contrast cardioprotective interventions in humans occur in the presence of various co-morbidities, such as arterial hypertension, obesity, diabetes, hypercholesterolemia, and often advanced age. Although there are numerous reports examining the role of adenosine and its receptors in these pathologies (Long et al., 2010; Wang et al., 2010; Bot et al., 2012; Koupenova et al., 2012; Sangsiri et al., 2013; Zhang et al., 2013; Teng et al., 2014; Nayak et al., 2015; Yang et al., 2016), the only one of these areas in which adenosine cardioprotection has been examined is in healthy aged rats and mice, and results have been conflicting. Isolated heart studies in 16–18 month old mice indicate a loss in the ability of adenosine and the A_1_ and A_3_ adenosine receptor agonists CPA and Cl-IB-MECA to reduce ischemia-reperfusion injury (Headrick et al., 2003; Peart et al., 2014). Two studies in rat hearts have led to contradictory findings as Schulman et al. (2001) reported a loss of adenosine A_1_ agonist (CCPA) preconditioning in 18–20 month-old Wistar rats, whereas Kristo et al. (2005) reported enhanced infarct size reduction in 24–26 months Fischer 344 × Brown Norway hybrid (F344 × BN) rats with the mixed (A_1_ and A_2A_) agonist AMP579. A major difference in these rat studies is that the former was conducted in an isolated perfused heart preparation, whereas the latter was conducted *in vivo*, where the effects of the circulating agonist continued into reperfusion. Interestingly in both rat and mouse studies the effects of A_1_ and A_2A_ agonists on heart rate and coronary flow in aged hearts were similar to those in young adult hearts. These findings are consistent with the reports of unaltered A_2A_ agonist (regadenoson) increases in coronary flow in aged human hearts (Cerqueira et al., 2008). One of the major limitations in the field of adenosine receptor cardioprotection is the lack of studies in animal models with clinically relevant morbidities. It is likely that these co-morbidities will alter myocardial adenosine receptor subtype expression and/or signaling mechanisms, as has been reported in models of atherosclerosis and diabetes (Long et al., 2010; Cabiati et al., 2015).

## Treatment paradigms

Another deficiency in the literature, related to current experimental studies, are the experimental treatment paradigms that are typically used. The majority of experimental studies on adenosine A_1_ and A_3_ receptors, as well as several studies A_2B_ receptors, have involved administration of agonists prior to ischemia to reduce ischemia-reperfusion injury (Headrick and Lasley, 2009; McIntosh and Lasley, 2012). Such a treatment paradigm has relevance to open heart surgery and preservation solutions for cardiac transplantation, but there have been no clinical trials to date, even assessing safety, for the use of adenosine receptor agonists. In contrast experimental studies on adenosine A_2A_ and A_2B_ agonists have focused on reperfusion treatments for the reduction of myocardial infarct size (Headrick and Lasley, 2009; McIntosh and Lasley, 2012), which upon initial review would appear to have some clinical relevance. Initial animal studies with adenosine A_2A_ agonist reperfusion treatment, which were all successful, occurred soon after the controversial experimental results with adenosine (Olafsson et al., 1987; Homeister et al., 1990; Goto et al., 1991; Pitarys et al., 1991; Vander Heide and Reimer, 1996) and the equivocal results of the acute myocardial infarction study of adenosine (AMISTAD) trial published in 1999 (Mahaffey et al., 1999).

However, the vast majority of experimental studies have initiated treatments during late ischemia or at the onset of reperfusion. Such early reperfusion treatments in the setting of acute myocardial infarction (MI) are not feasible given the time involved from the onset of patient symptoms to the diagnosis of MI and initiation of reperfusion therapy. In fact the results of the AMISTAD-II trial indicated that patients [particularly those receiving thrombolytic therapy, rather than percutaneous coronary intervention (PCI)] receiving intravenous adenosine within 3 h of symptom onset showed significantly reduced 1 and 6 months mortality compared with placebo (Kloner et al., 2006). Patients obtaining adenosine reperfusion treatment later than 3 h of symptom onset received no beneficial effect. These clinical observations were similar to those by (Toufektsian et al., 2006), who reported that a 1 h delay in the reperfusion administration of the highly selective A_2A_ agonist, ATL146e, failed to reduce 24 h infarct size in mice (Toufektsian et al., 2006). Interestingly, the delayed treatment did increase post-MI cardiac function and reduce inflammation. We previously reported (Lasley et al., 2001) that an intracoronary infusion of the A_2A_ agonist CGS21680, 2 h after reperfusion, increased regional preload-recruitable stroke work and stroke work area (load-insensitive parameters of cardiac contractility) in a porcine model of reversible myocardial ischemia-reperfusion injury. This effect was determined to be independent of effects on coronary blood flow, and the same infusion in normal myocardium had no effect on regional contractile function. In summary, the lack of experimental studies in clinically relevant models with appropriate treatment protocols, is likely to have contributed to the lack of clinical trials examining the efficacy of adenosine or receptor agonists for treating acute MI. The primary focus on reduction of acute injury is not only inconsistent with clinical trends of more patients living with chronic myocardial ischemia, but it also neglects some of the other recognized beneficial effects of adenosine receptors.

## Chronic myocardial ischemia models

Another weakness in experimental models of adenosine receptor cardioprotection is the primary focus on acute cardioprotection. This is an inherent limitation of *in vitro* models, but this continues to be a significant weakness of *in vivo* models, in which the study endpoint is typically infarct size after 2–3 h of reperfusion, with only a limited number of studies extending reperfusion to 24 h. Data from the National Heart, Lung and Blood Institute (NHLBI) and other sources indicate that over the past 40 years, deaths from acute MI have decreased significantly, whereas the incidence of heart failure and deaths from heart failure have increased (Krumholz et al., 2009). Experimental studies with short durations of reperfusion exclude significant components of the post-ischemic inflammatory process, which is a primary contributor to post-MI ventricular remodeling and subsequent heart failure. Such studies also exclude the well-known modulatory effects of adenosine receptors on inflammatory processes. It is also well-recognized that adenosine receptor expression and adenosine formation are increased in chronic inflammation, similar to that seen in chronically ischemic hearts (Xaus et al., 1999; Sun et al., 2006; Hasko et al., 2008; Feoktistov and Biaggioni, 2011; Belikoff et al., 2012).

Unfortunately, there have only been a very limited number of experimental studies assessing the cardioprotective effects of adenosine and/or receptor agonists in chronic models of myocardial ischemia. In what appears to be the first such study, Villarreal et al. (2003) reported that a 2 h intravenous infusion of an adenosine kinase inhibitor, which increases endogenous adenosine levels, but not adenosine itself, in rats just prior to reperfusion (following a 2 h occlusion) increased 2 week post-MI ischemic zone wall thickness, consistent with reduced ventricular remodeling (Villarreal et al., 2003). Wakeno et al. subsequently reported that a 3 weeks treatment (twice daily intraperitoneal injections) with the non-selective agonist, 2-chloroadenosine, starting at 7-day post-MI in rats reduced cardiac remodeling and cardiac fibrosis (Wakeno et al., 2006). Based on results with multiple adenosine receptor antagonists, the authors concluded that this protective effect was due to adenosine A_2B_ receptor stimulation. More recently Sabbah et al. reported that chronic treatment with a partial A_1_ receptor agonist (capadenoson), in a coronary microembolization-induced model of heart failure in canines, improved left ventricular function, decreased fibrosis, and reduced plasma n-terminal pro-brain natriuretic peptide concentrations (Sabbah et al., 2013). These beneficial effects observed in the absence of changes in heart rate, blood pressure, or renal function, but were accompanied by increased expression of left ventricular sarcoplasmic reticulum calcium ATPase activity, mitochondrial uncoupling proteins (UCP) and glucose transporters. These latter observations suggest that capadenoson's beneficial effects were due to direct effects on the heart, although the specific mechanism remains unknown.

Despite the lack of chronic experimental myocardial ischemia studies, there is evidence suggesting that adenosine may exert beneficial effects in patients with chronic myocardial ischemia. Bulluck et al. (2016) conducted a meta-analysis on the results of 13 randomized clinical trials using intracoronary or intravenous adenosine in patients with ST-segment elevation MI (STEMI). They concluded that intracoronary adenosine therapy in the presence of primary percutaneous coronary intervention (PPCI) was effective in reducing post-STEMI heart failure, but not in terms of other end-points such as death, non-fatal MI, or revascularization. They also concluded that these effects were most likely due to infarct size reduction via less reperfusion injury, although they could not exclude a role in reducing ventricular remodeling. A double blinded, placebo controlled Phase 2 clinical trial (7 days of treatment) with the A_1_ partial agonist BAY1067197 (neladenoson bialanate) in patients with heart failure with reduced ejection fraction indicated that this agent was safe, although no beneficial effects on cardiac function were observed (Voors et al., 2017).

The possibility that adenosine receptors may modulate post-MI remodeling in patients highlights/emphasizes the need to conduct clinically relevant experimental chronic studies, rather than acute studies. Cardiac remodeling is due to chronic inflammation and fibrosis, mediated by immune cells and fibroblasts, both of which express at least two adenosine receptors. Adenosine A_2A_ receptors are well recognized for their anti-inflammatory effects, and there are reports that A_2B_ receptors may exert both anti- and pro-inflammatory effects (Hasko et al., 2008; Csoka et al., 2010; Feoktistov and Biaggioni, 2011; Linden, 2011; Haskó and Cronstein, 2013). Likewise, there is evidence that both A_2A_ and A_2B_ receptors regulate fibroblast function, including cardiac fibroblasts, although there are conflicting reports on their specific effects (Zhong et al., 2005; Villarreal et al., 2009; Zhang et al., 2014; Karmouty-Quintana et al., 2015; Shaikh and Cronstein, 2016). These observations and reports of adenosine receptor involvement in various chronic diseases (Long et al., 2010; Wang et al., 2010; Bot et al., 2012; Koupenova et al., 2012; Sangsiri et al., 2013; Zhang et al., 2013; Teng et al., 2014; Nayak et al., 2015; Yang et al., 2016), clearly warrant more studies on adenosine receptor modulation of chronic myocardial ischemia.

## Receptor agonists or antagonists?

One aspect related to the lack of studies on adenosine receptor modulation of chronic myocardial ischemia, which has only recently been recognized, is the issue of receptor agonism vs. antagonism. All studies examining acute myocardial ischemia models have focused on treatment with receptor agonists, and in these studies blockade or deletion of any of the four adenosine receptor subtypes has resulted in little exacerbation of ischemia-reperfusion injury. However, there are numerous reports that antagonism or deletion of adenosine receptor subtypes is protective in models of both arterial and pulmonary hypertension, pulmonary and renal fibrosis, and sepsis (Sun et al., 2006; Kolachala et al., 2008; Zhou et al., 2011; Belikoff et al., 2012; Karmouty-Quintana et al., 2012, 2013a; Zhang et al., 2013; Nayak et al., 2015). The majority of these studies have focused on the anti-inflammatory and/or anti-fibrotic effects of A_2B_ receptor blockade.

At first glance, reports that A_2B_ antagonism is beneficial in chronic disease would appear to be contradictory to the reports of beneficial effects of A_2B_ agonism in the acute phase of myocardial ischemia. However, a review of the literature indicates there is substantial evidence for adenosine receptors playing dual roles in acute vs. chronic pathologies. For example, although the anti-inflammatory role of A_2A_ receptors has been recognized for years, there are more recent reports that A_2A_ receptor stimulation prolong IL-1β release and caspase-1 activity, consistent with inflammasome activation, in murine macrophages (Ouyang et al., 2013) and brain (Chiu et al., 2014). Ingwersen et al. (2016) also reported that while A_2A_ receptor stimulation was acutely beneficial in a murine model of autoimmune neuroinflammation, chronic inflammation was reduced in A_2A_ KO mice (Ingwersen et al., 2016). The time-dependent, opposing effects of adenosine A_2A_ and A_2B_ receptors are in fact consistent with the dual role of inflammation in the post-ischemic heart. Macrophages participate in both the initial pro-inflammatory phase to remove dead and dying tissue, but the subsequent anti-inflammatory period is necessary in order for cardiac fibroblasts to differentiate into myofibroblasts, which then deposit collagen (Nahrendorf et al., 2010; Frangogiannis, 2012; Prabhu and Frangogiannis, 2016). Collagen deposition is critical for maintaining scar thickness and strength in the infarct zone, but prolonged inflammation and excess collagen deposition lead to adverse ventricular remodeling (Nahrendorf et al., 2010; Frangogiannis, 2012; Prabhu and Frangogiannis, 2016).

To date there are only a very limited number of studies assessing potential beneficial effects of adenosine receptor blockade in chronic heart disease, both in experimental models and in clinical trials. Beneficial effects of A_1_ receptor antagonists in experimental models of acute heart failure have been reported going back well over a decade (Nagashima et al., 1995; Givertz et al., 2007; Greenberg et al., 2007; Slawsky and Givertz, 2009). These beneficial effects were thought to be due primarily to blockade of A_1_ receptor-mediated vasoconstriction of the renal afferent artery as well as proximal tubule reabsorption of sodium. Despite these positive findings in initial small trials, a large, randomized, placebo controlled Phase 3 trial (PROTECT) with the A_1_ antagonist, rolofylline, failed to significantly impact cardiac or renal primary or secondary end points (Massie et al., 2010). More recently, the beneficial effects of A_2B_ receptor antagonism in experimental models of post-MI remodeling have been reported. Toldo et al. (2012) reported that the administration of the A_2B_ antagonist, GS-6201, immediately following a permanent occlusion in mice resulted in a thicker scar, less LV hypertrophy and improved post-MI cardiac function after 4 weeks. Similar findings were reported with the same A_2B_ antagonist in a chronic occlusion-reperfusion model in rats (Zhang et al., 2014). Thus, despite numerous experimental studies reporting beneficial effects of adenosine receptor antagonists in multiple non-cardiac pathologies, there remains a paucity of similar studies in chronic heart disease.

## Additional unresolved issues

In addition to the above clinical-relevance issues, there are some unresolved basic science matters related to adenosine receptor-mediated cardioprotection. Adenosine receptors are differentially expressed on multiple cells types, thus altering the tissue and organ response to even selective agonists or antagonists (Chen et al., 2013; Sheth et al., 2014). This may explain, in part, the reported time-dependent differences in acute vs. chronic effects of adenosine receptor antagonists (Karmouty-Quintana et al., 2013b). As stated earlier, all four adenosine receptors have been implicated in protection against acute myocardial ischemia-reperfusion injury, and there are multiple reports that this is mediated by all four receptor subtypes modulating the same signaling pathways, presumably in cardiomyocytes (McIntosh and Lasley, 2012). There has yet to be an explanation why cardiomyocytes would express four different adenosine receptor subtypes exerting the same effect via the same signaling pathways. As discussed previously, the lack of studies addressing adenosine receptor effects in models of chronic myocardial ischemia have hindered our knowledge of the roles of specific adenosine receptors in non-cardiomyocytes, such as endothelial cells, immune cells and cardiac fibroblasts. Species-dependent differences in the selectivity of adenosine receptor agonists and antagonists have been recognized for many years, and this topic has most recently been addressed by Alnouri et al. (2015) and Jacobson and Müller (2016). This issue has undoubtedly had effects on the interpretation of numerous experimental studies, but its biggest impact has probably been on clinical trials, where despite reports of safety and tolerance, there remain few reports on the efficacy of adenosine receptor analogs in treating ischemic heart disease (Massie et al., 2010; Voors et al., 2017). Finally, there is increasing evidence in multiple tissues, that adenosine receptors may exert their effects, in part, via receptor dimerization (Zhan et al., 2011; McIntosh and Lasley, 2012; Chandrasekera et al., 2013; Chen et al., 2013). This aspect of adenosine receptor modulation of myocardial ischemia-reperfusion injury needs to be further explored, as this may lead to new, clinically relevant therapies.

In conclusion, nearly 30 years of experimental findings support the hypothesis that adenosine receptors modulate acute myocardial ischemia-reperfusion injury. Despite this evidence, the use of adenosine, adenosine modulators, or adenosine analogs for treatment of cardiac injury has not been accepted clinically, nor have there been many clinical trials. Clearly the next phase of research on adenosine receptor cardioprotection needs to establish the role of adenosine receptor agonists and antagonists in more clinically relevant models of myocardial ischemia.

## Author contributions

The author confirms being the sole contributor of this work and approved it for publication.

### Conflict of interest statement

The author declares that the research was conducted in the absence of any commercial or financial relationships that could be construed as a potential conflict of interest.
